# Fish Intake in Relation to Fatal and Non-Fatal Cardiovascular Risk: A Systematic Review and Meta-Analysis of Cohort Studies

**DOI:** 10.3390/nu15214539

**Published:** 2023-10-26

**Authors:** Hannah Ricci, Maddalena Gaeta, Carlotta Franchi, Andrea Poli, Maurizio Battino, Alberto Dolci, Daniela Schmid, Cristian Ricci

**Affiliations:** 1 Africa Unit for Transdisciplinary Health Research (AUTHeR), North-West University, Potchefstroom 2531, South Africa; 2 Department of Public Health, Experimental and Forensic Medicine, University of Pavia, 27100 Pavia, Italy; 3 Laboratory of Pharmacoepidemiology and Human Nutrition, Department of Health Policy, Istituto di Ricerche Farmacologiche Mario Negri IRCCS, 20156 Milan, Italy; carlotta.franchi@marionegri.it; 4 Italian Institute for Planetary Health (IIPH), 20124 Milan, Italy; 5 Nutrition Foundation of Italy (NFI), 20124 Milan, Italy; poli@nutrition-foundation.it; 6 Department of Clinical Sciences, Università Politecnica delle Marche, 60126 Ancona, Italy; m.a.battino@staff.univpm.it; 7 International Research Center for Food Nutrition and Safety, Jiangsu University, Zhenjiang 212013, China; 8 Research Group on Food, Nutritional Biochemistry and Health, Universidad Europea del Atlántico, 39011 Santander, Spain; 9 Sustainable Development Department, Bolton Food SpA, 20124 Milan, Italy; adolci@boltonfood.com; 10 Division for Quantitative Methods in Public Health and Health Services Research, Private University of Health Sciences, Medical Informatics and Technology, 6060 Hall, Austria; daniela.gallmetzer@umit-tirol.at

**Keywords:** fish, fatty fish, nutrition, cardiovascular risk, cardiovascular mortality, systematic review, meta-analysis, meta-regression, cohort study

## Abstract

Epidemiological studies have shown that eating fish significantly reduces cardiovascular disease (CVD) incidence and mortality. However, more focused meta-analyses based on the most recent results from prospective cohort studies are needed. This systematic review and meta-analysis aims to update the association between fish intake and cardiovascular disease (CVD) risk using recent prospective studies. A systematic review and meta-analysis following the PRISMA guideline was conducted based on a random effects synthesis of multivariable-adjusted relative risks (RRs) of high vs. low categories of fish intake in relation to CVD incidence and mortality. Non-linear meta-regression was applied to investigate the shape of the association between fish intake and CVD risk. Sensitivity analysis and stratifications by type of CVD outcome, type of fish intake and type of cooking were performed. Based on 18 papers reporting 17 independent estimates of CVD risk (1,442,407 participants and 78,805 fatal and non-fatal CVD events), high vs. low intake of fish corresponded to about 8% reduced CVD risk (RR = 0.93 [0.88–0.98]). According to a non-linear dose–response meta-regression, 50 g of fish intake per day corresponded to a statistically significant 9% reduced fatal and non-fatal CVD risk (RR = 0.92 [0.90–0.95]). Similarly, fish intake in the range of a weekly intake of two to three portions of fish with a size of 150 g resulted in 8% fatal and non-fatal CVD risk reduction (RR = 0.93 [0.91–0.96]). The recommended two portions of fish a week reduces the risk of CVD outcomes by approximately 10%. A full portion of fish a day reduces CVD risk by up to 30%.

## 1. Introduction

Globally, 41 million people (74% of all deaths) are estimated to die from non-communicable diseases (NCDs) every year with 17 million of these deaths occurring before 70 years of age [1]. Among all NCD-related mortality and incidence, cardiovascular diseases (CVDs) account for an estimated 523 million prevalent cases and 18.6 million deaths yearly, as well as an estimated 34.4 million years living with disability [1,2]. Among CVD cases, coronary heart disease (CHD) and stroke account for an estimated 197 million and 101 million prevalent cases, respectively. In 2019, the disability-adjusted life years (DALYs) due to CHD was estimated at 182 million while that of stroke was estimated at 143 million, with deaths due to CHD and stroke being estimated at 9.14 million and 6.55 million, respectively [1,2]. Among the established risk factors [3], the most acknowledged and modifiable for CVDs are unhealthy diets and lifestyles [1,3,4]. Epidemiological studies have consistently shown that eating a moderate balanced diet containing potentially healthy foods such as fish can significantly help reduce CVD incidence and mortality [4]. For example, fatty fish such as sardine, salmon, trout and mackerel contain high amounts of long-chain omega-3 polyunsaturated fatty acids (ω-3 PUFA), namely docosahexaenoic acid (DHA; 22:6n-3) and eicosapentaenoic acid (EPA; 20:5n-3), which may reduce the risk of CVD [5,6,7]. Additionally, prospective studies have reported an association between fish consumption and CVD events [8,9,10,11]. In a systematic review and meta-analysis by Giosuè and colleagues, a high intake of fatty fish was associated with a 9% reduced risk of CVD [9]. A systematic review and meta-analysis by Bechthold et al. [8] showed that dietary intake of fish was associated with 14%, 16% and 25% reduced risk of CHD, stroke and heart failure, respectively. These findings agree with two other systematic reviews and meta-analyses that found that for every 20 g per day increase in fish intake or 80 mg per day increased intake of marine n-3 PUFAs, a 4% reduced risk of CHD incidence and mortality was observed [10,11]. Additionally, evidence on fish oil supplement intake and CVD risk reduction has been well established by means of randomised controlled studies and meta-analyses [12,13]. Although evidence of the association between fish intake, reduced CVD risk and mortality continues to increase, more focused meta-analyses based on the most recent results from prospective cohort studies are needed. Notably, results from prospective cohort studies can provide evidence of higher methodological quality compared with studies with retrospective or cross-sectional designs, which may be affected by certain biases, such as recall bias and reversal causation [14,15,16]. To inform current nutrition guidelines, more information about the effects of type of fish and cooking method on the various CVDs is needed. Against this background, the aim of this systematic review and meta-analysis was to update the association between different types of fish intake, considering various cooking methods, and incidence of and mortality from different CVDs using prospective study data captured from 2012 until 2023. We also aimed to provide an update on the dose–response relationship between fish intake and fatal and non-fatal CVD risk.

## 2. Methods

This systematic review and meta-analysis followed the Preferred Reporting Items for Systematic Reviews and Meta-Analyses (PRISMA) guidelines [17]. The PECO(ST) approach was used to conduct the paper selection [18]. Each PECO(ST) item was defined using a hierarchical strategy based on Medical Subject Headings (MeSH). The target population (P) was defined as healthy adults of either sex. Studies were included if they considered fish intake of any kind as an exposure (E) and if they provided risk estimates for the comparison (C) of high vs. low categories of fish intake (for example, centiles, servings/weeks, etc.). Any type of CVD or CVD-related mortality was considered as the outcome (O). Finally, only observational studies with a prospective study design (S) published in peer-reviewed journals between 1 January 2012 (T) and 31 March 2023 were included. We excluded studies not yet published in their final form, and those for which the full article was not available in English. Meta-analyses and systematic reviews, reports, patents, theses, posters, conference presentations, letters, opinion papers and seminar papers were also excluded. The MeSH search terms within any of the PECO(ST) items mentioned above were linked u the OR operator. The AND operator was used to link the search strings from any of the PECO(ST) items. The full search strategy is provided in Supplementary Material S1. Selection of eligible papers was performed in duplicate by two independent researchers (HR and MG). The full text articles were read independently by the same authors for final inclusion. Any disagreement about study inclusion was solved by consensus or by consulting a third senior author (CR or DS).

### 2.1. Information Sources

The systematic literature search was conducted using the database of the North-West University with more than 100 literature repositories, including MEDLINE-PubMed, Cochrane and CINAHL. The same search string was used to search Embase, which is not included in the above-mentioned repository. In addition, reference lists of previously published systematic reviews and meta-analyses were investigated for eligible studies.

### 2.2. Assessment of Study Quality and Risk of Bias

Two independent authors (HR and MG) used the Newcastle Ottawa Scale (NOS) to assess the risk of bias and overall study quality [19]. The NOS is a tool validated to identify potential bias and overall quality in observational studies. Up to nine points can be awarded to cohort studies using the NOS scale, four points can be awarded for the selection of the study participants, two points can be awarded for the comparability of exposed and not-exposed cohorts and up to three points for the assessment of outcome and for the adequacy of follow-up. We assumed that studies with an NOS score greater or equal to six were of moderate to high quality. Any disagreement about the assessment of study quality was solved by consensus or by consulting a third senior author (CR, MG or DS).

### 2.3. Statistical Analyses

Our main meta-analysis was based on a random effects synthesis of multivariable-adjusted relative risks (RRs) of high vs. low categories of fish intake in relation to CVD incidence and mortality. In this model, the study’s weight was given using inverse variance, with the weight of the *i*-th study being computed as w_i_ = 1/(s_i_^2^ + t^2^), where s_i_^2^ was the variance of the *i*-th study and t^2^ was the overall variance. The fixed effect estimate was also illustrated using forest plots. In general, the included studies contributing to the meta-analyses had one risk estimate only. Results from a single study were pooled using a fixed effect meta-analysis when more than one risk estimate was provided, for example, for sex, or for specific types of fish intakes or CVD. Furthermore, we chose the study with the highest number of CVD cases when different studies or papers provided data from the same cohort. Results were kept separate if results from independent cohorts were reported by a single paper. The Cochrane Q test (if at least 10 studies were included) and the I^2^ statistic (considering I^2^ > 50% for substantial heterogeneity) were reported to show between-study heterogeneity. Between-study heterogeneity was investigated using stratification analyses and linear meta-regression. Sensitivity analyses were performed omitting one study at a time, and potential publication bias was detected using visual inspection of the funnel plot and using Egger’s test [20]. Finally, the shape of the relationship between fish intake and CVD risk was assessed using a non-linear dose–response meta-regression based on restricted cubic splines with 5th, 35th, 65th and 95th percentiles of fish intake as knots [21]. All statistical analyses were performed using the STATA software version 14. The METAN, METANINF, METABIAS, METAFUNNEL and METAREG packages were used to conduct the random and fixed meta-analyses of the RR estimates, sensitivity analyses, Egger’s test, funnel plots and linear meta-regression, respectively. The GLST function was used to perform the restricted cubic spline non-linear meta-regression. All statistical tests were conducted at a significance level of 5% (α = 0.05), except for Egger’s test, where a significance level of 10% (α = 0.1) is recommended [22].

## 3. Results

**Figure 1:**
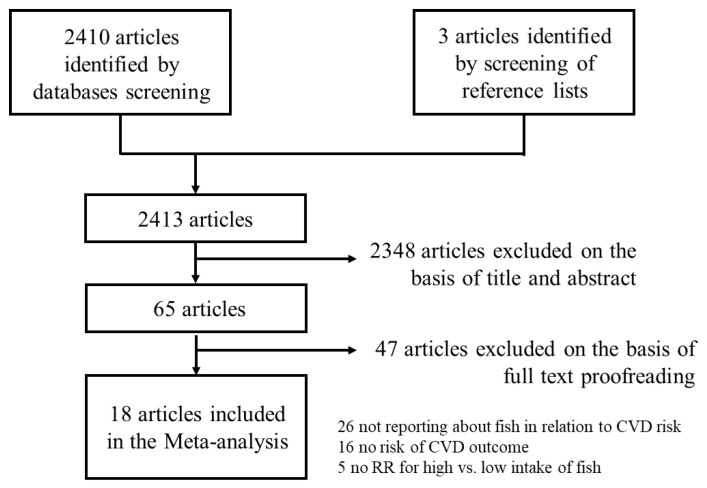
Flowchart of paper selection.

Our search string resulted in 2410 records with three additional papers identified by screening the reference list of included systematic reviews and meta-analyses, resulting in a total of 2413 records. A total of 2348 records were excluded after reading the title and abstract, leaving 65 records. Of the remaining articles, 18 were included as they met our inclusion criteria [23,24,25,26,27,28,29,30,31,32,33,34,35,36,37,38,39,40]. Because one paper [34] reported results from three separate studies, our systematic review and meta-analysis was based on 20 independent studies. The flowchart of paper selection is shown in Figure 1. Among the included studies, 10 were conducted in Europe [23,24,25,26,27,28,29,32,33,39]. Specifically, two studies were from the Netherlands [28,39] and two were from Sweden [25,33]. Denmark [27], Germany [32], Italy [26] and Spain [23] contributed one study each. One study was based on the large EPIC cohort study conducted in many European countries [29]. Four of the included studies were from the Asia-Pacific region with two studies from Japan [30,31] and one study each from China [38] and Australia [36]. Three studies were from the USA [35,37,40] and the remaining three studies were multinational [34]. The median age of the participants was 56.3 years, ranging between 20 and 83 years. One study included only women [37] while one included only men [24]. The median percentage of men was 45.2% (range = 25.2% to 70.3%). The median sample size was 36,713 (range = 3202 to 421,309) participants with a median follow-up of 12.2 (range = 2.2 to 24.6) years, resulting in a median of 589,335 (range = 8701 to 6,070,000) person-years. The median number of both fatal and non-fatal events was 1865 (range = 277 to 29,648); that of non-fatal events was 1532 (range = 353 to 8201) and fatal events was 1135 (range = 117 to 29,648). The most common CVD reported was myocardial infarction (MI) with a median of 1123 (range = 440 to 3806) cases, followed by stroke (median = 674 cases, range 66 to 3925) and CHD (median = 307 cases, range 287 to 2134). The median NOS score was 7 (range = 5 to 8) with all but two studies [35,39] having a NOS score above or equal to 6. The characteristics of the included studies are presented in Table 1.

### 3.1. Meta-Analysis of High vs. Low Intake of Fish in Relation to CVD Risk

**Figure 2:**
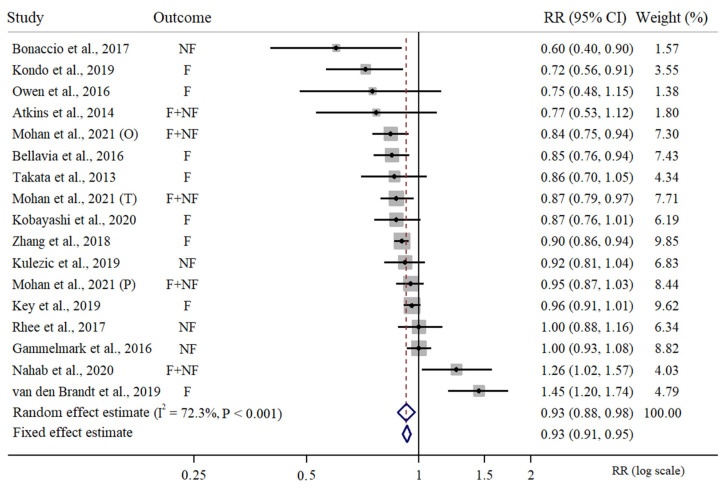
Random effects meta-analysis of relative risk of high vs. low intake of fish in relation to non-fatal and fatal CVD risk. Three different studies were included from the paper of Mohan et al., 2021: Origin (O), PURE (P) and On Target and Transcend (T) [24,25,26,27,29,30,31,33,34,35,36,37,38,39,40].

**Figure 3:**
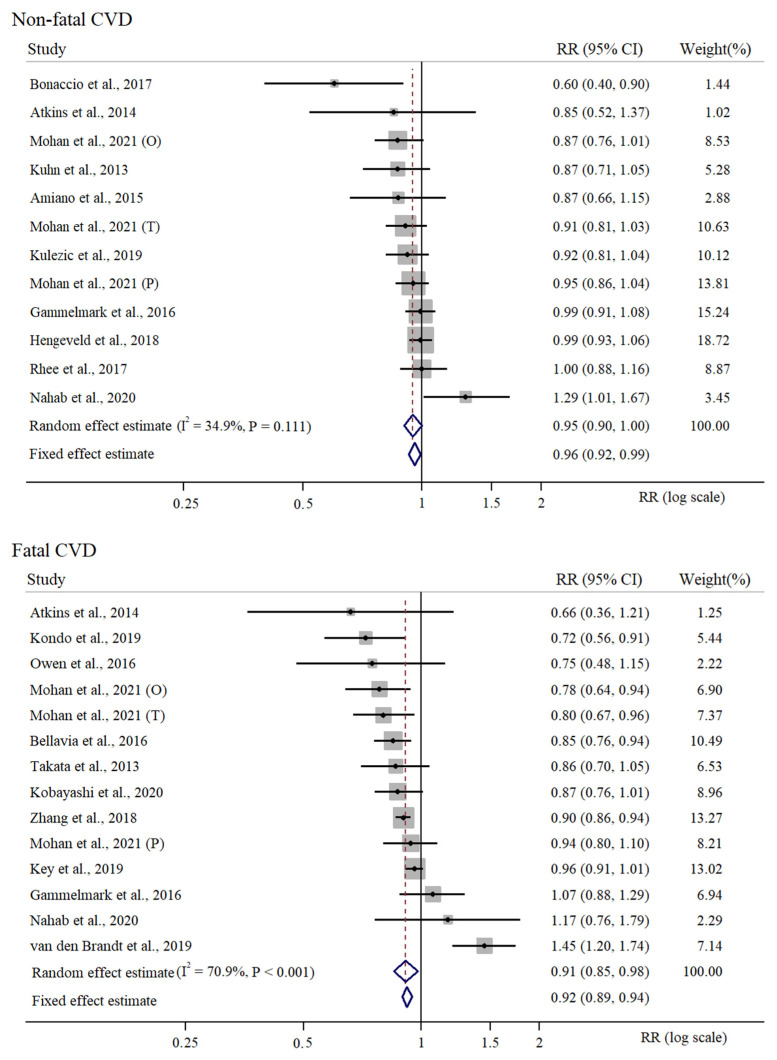
Random effects meta-analysis of relative risk of high vs. low intake of fish by non-fatal and fatal CVD risk. Three different studies were included from the paper of Mohan et al., 2021, Origin (O), PURE (P) and On Target and Transcend (T) [24,25,26,27,28,29,30,31,32,33,34,35,36,37,38,39,40].

There were 17 independent risk estimates of high vs. low intake of fish included in the meta-analysis considering any type of fish intake and any type of CVD outcome [24,26,27,29,31,33,34,35,36,37,39,40]. This meta-analysis was based on a cumulative sample size of 1,442,407 participants, 18,926,486 cumulative person-years and 78,805 CVD events. We estimated that high vs. low intake of fish would correspond to about 8% reduced risk of CVD (RR = 0.93, 95% CI = 0.88 to 0.98). In this meta-analysis, we observed a medium to large between-study heterogeneity (I^2^ = 72.3%) and a statistically significant Cochrane Q test (PQ-Cochrane < 0.001). The meta-analysis of the RR estimates of fatal and non-fatal CVD risk for high vs. low intake of fish is presented in Figure 2. There were 12 independent estimates considered for the meta-analysis of RR for high vs. low intake of fish in relation to non-fatal CVD risk [23,24,26,27,28,32,33,34,35,37], resulting in 478,053 participants, 5,810,375 person-years and 28,396 non-fatal CVD events. We estimated a 5% non-fatal reduction in CVD risk for high vs. low intake of fish along with a non-significant between-study heterogeneity (RR = 0.95, 95% CI = 0.90 to 1.00, I^2^ = 34.9%, PQ-Cochrane = 0.111). Finally, we considered 14 independent RR estimates for fatal CVDs in relation to high vs. low fish intake [24,25,27,29,30,31,34,35,36,38,39,40]. According to this meta-analysis, based on 1,356,036 participants, 17,340,624 person-years and 55,676 fatal CVD cases, we observed an overall 10% reduced fatal CVD risk for high vs. low intake of fish, along with a significant between-study heterogeneity (RR = 0.91, 95% CI = 0.85 to 0.98, I^2^ = 70.9%, PQ-Cochrane < 0.001). The meta-analysis of the RR estimates of fatal and non-fatal CVDs for high vs. low intake of fish is presented in Figure 3.

### 3.2. Supplementary Meta-Analysis of High vs. Low Intake of Fish in Relation to CVD Risk

Three studies provided data on intake of fatty fish [26,27,29]. We estimated a 12% reduced risk of any fatal or non-fatal CVD for high vs. low intake of fatty fish, along with a non-relevant between-study heterogeneity (RR = 0.89, 95% CI = 0.79 to 1.00, I^2^ = 57.9%). By comparison, based on the same included studies, a reduced fatal and non-fatal CVD risk was not observed when looking at high vs. low intake of lean fish (RR = 1.05, 95% CI = 0.99 to 1.13, I^2^ = 0%). Two studies considered CVD risk in relation to intake of fried fish [35,40]. Here, we observed a statistically significant 3% increased fatal and non-fatal CVD risk for high vs. low intake of fried fish (RR = 1.03, 95% CI = 1.00 to 1.07, I^2^ = 0%). Also, based on three studies [35,37,40], high vs. low intake of non-fried fish was not associated with fatal or non-fatal CVD risk (RR = 0.94, 95% CI = 0.65, 1.36, I^2^ = 83%). When looking at specific CVD outcomes, six studies [27,28,32,34] investigated the association between intake of fish and MI. No statistically significant association was observed for high vs. low intake of fish in relation to fatal and non-fatal MI (RR = 0.96, 95% CI = 0.90 to 1.03, I^2^ = 18.5%). Stroke risk in relation to fish intake was investigated by seven studies [23,26,28,32,34]. According to this meta-analysis, no statistically significant association exists between high vs. low intake of fish and fatal and non-fatal stroke risk (RR = 0.95, 95% CI = 0.86 to 1.05, I^2^ = 27.0%). This result was confirmed when looking at specific types of stroke. Four studies [28,32,35,38] investigated ischaemic stroke risk in relation to fish intake (RR = 0.95, 95% CI = 0.86 to 1.05, I^2^ = 27.0%) and three studies [28,32,38] investigated haemorrhagic stroke in relation to fish intake (RR = 0.91, 95% CI = 0.68 to 1.23, I^2^ = 33.5%). Finally, no statistically significant association was observed when considering high vs. low intake of fish in relation to CHD risk (RR = 0.92, 95% CI = 0.64 to 1.33, I^2^ = 49.5%). The three studies included in the paper of Mohan et al. [34] reported data about sudden CVD mortality risk in relation to fish intake. The random effects meta-analysis resulted in a borderline non-significant 22% reduced sudden CVD mortality risk (RR = 0.82, 95% CI = 0.67 to 1.02, I^2^ = 0%).

### 3.3. Dose–Response Analysis of Fish Intake in Relation to Fatal and Non-Fatal CVD Risk

**Figure 4:**
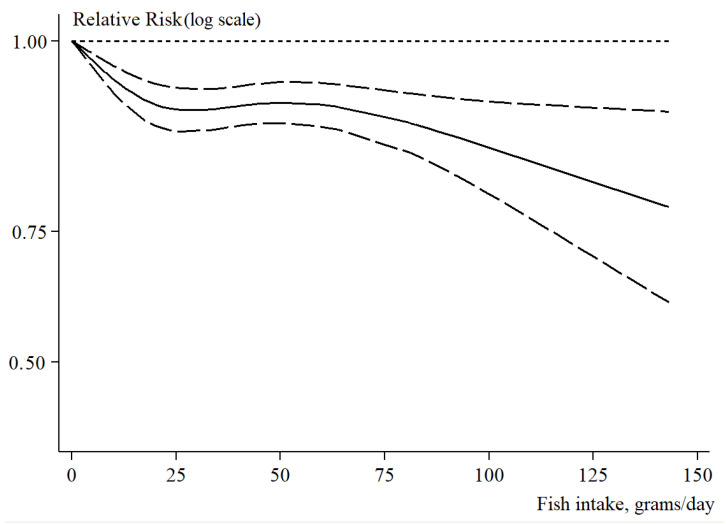
Non-linear dose–response analysis of fish intake (g per day) vs. non-fatal and fatal CVD risk; dotted lines portray 95% confidence limits.

There were 10 studies included in the analysis of non-linear dose–response meta-regression of fish intake in relation to fatal and non-fatal CVD risk [23,26,28,31,32,34,38,40]. In this analysis, we observed a monotonic decreasing S-shaped curve that portrayed a non-linear relationship between fish intake and fatal and non-fatal CVD risk (P for non-linearity < 0.001). We observed that even a relatively small fish intake corresponded to a reduced fatal and non-fatal CVD risk. Moreover, we estimated that 50 g of fish intake per day would correspond to a statistically significant 9% reduced fatal and non-fatal CVD risk (RR = 0.92, 95% CI = 0.90 to 0.95). Similarly, fish intake in the range of a weekly intake of two to three portions of fish with a size of 150 g would result in 8% fatal and non-fatal CVD risk reduction (RR = 0.93, 95% CI = 0.91 to 0.96). A further increase in fish intake corresponded to a larger reduction in fatal and non-fatal CVD risk. For instance, a fish intake of 100 to 150 g a day corresponded to a 16% (RR = 0.86, 95% CI = 0.80 to 0.92) to 28% (RR = 0.78, 95% CI = 0.68 to 0.90) reduced fatal and non-fatal CVD risk, respectively. The non-linear dose–response meta-regression of fish intake in relation to fatal and non-fatal CVD risk is shown in Figure 4.

### 3.4. Sensitivity Analyses, Assessment of Publication Bias and Determinants of Heterogeneity

When excluding the studies from Van der Brandt et al. [39] and Kondo et al. [31], we observed RR estimates of high vs. low intake of fish in relation to fatal and non-fatal CVD risk ranging between 0.91 (95% CI = 0.87 to 0.95, I^2^ = 57.5%, P_Q-Cochrane_ = 0.002) and 0.94 (95% CI = 0.89 to 0.99, I^2^ = 71.9%, P_Q-Cochrane_ < 0.001), respectively. The exclusion of the two studies with increased CVD risk for higher fish intake and also lower NOS score [35,39] resulted in 11% reduced fatal and non-fatal CVD risk with reduced but still statistically significant between-study heterogeneity (RR = 0.90, 95% CI = 0.87 to 0.94, I^2^ = 48.3%, P_Q-Cochrane_ = 0.019). No evidence of publication bias emerged according to Egger’s test (P_Egger_ = 0.726) or by the visual inspection of the funnel plot (Supplementary Figure S1A). The same investigation conducted for non-fatal CVD risk in relation to fish intake confirmed the results observed above. Briefly, excluding one study at a time from the meta-analysis of non-fatal CVDs in relation to high vs. low intake of fish resulted in RR estimates ranging between 0.94 (95% CI = 0.89 to 1.00, I^2^ = 35.7%, P_Q-Cochrane_ = 0.114) and 0.96 (95% CI = 0.92 to 1.00, I^2^ = 14.9%, P_Q-Cochrane_ = 0.303), when excluding the studies by Hengeveld et al. [28] and Bonaccio et al. [26], respectively. After the exclusion of the study from Nahab et al. [35], we estimated 5% reduced non-fatal CVD risk for high fish intake and we observed a supplementary reduction in the between-study heterogeneity (RR = 0.95, 95% CI = 0.91 to 0.99, I^2^ = 12.5%, P_Q-Cochrane_ = 0.326). No evidence of publication bias was observed according to Egger’s test (P_Egger_ = 0.193) or visual inspection of the funnel plot (Supplementary Figure S1B). These results were confirmed by the sensitivity analyses when we excluded one study at a time. Notably, the risk estimates of fatal CVDs for high vs. low fish intake ranged between 0.89 (95% CI = 0.85 to 0.94, I^2^ = 41.4%, P_Q-Cochrane_ = 0.053) and 0.92 (95% CI = 0.86 to 0.99, I^2^ = 70.4%, P_Q-Cochrane_ < 0.001) when excluding the studies from Van der Brandt et al. [39] and Kondo et al. [31], respectively. We did not observe any evidence of publication bias based on Egger’s test (P_Egger_ = 0.765) and the visual inspection of the funnel plot (Supplementary Figure S1C).

When considering only those estimates adjusted for the overall energy intake, we observed a relevant reduction in the between-study heterogeneity (from 72.3% to 59.3%) and an RR estimate of fatal and non-fatal CVD risk for high vs. low intake of fish of 0.91 (95% CI = 0.87 to 0.95). We also observed a reduction in the between-study heterogeneity (from 72.3% to 48.3%) when considering only studies of higher methodological quality (NOS ≥ 6). Likewise, we observed an 11% reduced risk of fatal and non-fatal CVDs for high vs. low intake of fish (RR = 0.90, 95% CI = 0.87 to 0.94). The results from the stratified analyses are presented in Table 2. Finally, when looking at specific CVD outcomes, we observed that the exclusion of the On Target and Transcend (OTT) study [32] would result in a significant 8.7% stroke risk reduction in association with high fish intake (RR = 0.92, 95% CI = 0.84 to 0.99, I^2^ = 0%).

## 4. Discussion

A statistically significant risk reduction for fatal and non-fatal CVDs in association with fish intake was observed in this systematic review and meta-analysis. According to our analysis, we can estimate that high vs. low intake of fish would reduce CVD risk by a factor of up to 10%. This general result is broadly confirmed by the results from previous systematic reviews and meta-analyses [6,9,10,41,42]. Nevertheless, a reduced CVD risk in relation to fish intake was observed in the large majority of the included studies with two studies reporting a null association between fish intake and CVD risk [27,37] and two studies reporting an increased CVD risk for high intake of fish [35,39]. We observed that our results were robust with respect to the exclusion of the above-mentioned two studies reporting increased CVD risk for high fish intake. Notably, these apparently discordant results may reflect methodological factors, as these two studies could be defined as of medium to low methodological quality according to the NOS score. On the one hand, the oversampling of participants of low socioeconomic status in the study from Nahab et al. [35] may have resulted in a reverse causation phenomenon due to an overall low intake of fish. In this study, only about 12% of the oldest participants, those with higher CVD risk, had more than two servings of fish a week. On the other hand, we cannot exclude that the case-cohort design adopted in the study from Van der Brandt et al. [39] may have determined a biased association between fish intake and CVD risk.

Specific stratified meta-analyses were conducted to investigate the association of different types of fish intake with different types of CVD events. We confirmed results from a previous meta-analysis, reporting that high intake of fatty fish resulted in a statistically significant 12% reduced fatal and non-fatal CVD risk [9]. We also showed a statistically significant but slightly increased CVD risk for high intake of fried fish. Whereas the interpretation of reduced CVD risk in association with high intake of fatty fish is widely acknowledged due to high content of ω-3 PUFAs [12,43,44] in fatty fish, the interpretation of the higher CVD risk in relation to fried fish is more challenging. On the one hand, frying food with vegetable oils was not associated with increased CVD risk in a previous systematic review [45]. On the other hand, the use of specific types of vegetable oil such as palm oil might theoretically be associated, due to their high saturated fatty acid content, with an increased CVD risk, especially MI [46]. The association between saturated fatty acids and MI has been discussed in a recent meta-analysis [47], so the possibility that a high fried fish intake is just a marker of an unbalanced diet cannot be ruled out. We did not observe a statistically significant association between fish intake and any specific CVD outcomes. Nonetheless, a significant 9% risk reduction associated with a high fish intake was observed for stroke after we performed a sensitivity analysis excluding the results of the OTT study [34]. This evidence is novel, even if it was reported by previous studies on marine fatty intake in relation to stroke [48,49,50].

Our dose–response analysis provides certain useful information in terms of public health recommendations about fish intake. First, our results support the concept that even a small intake of fish is beneficial to cardiovascular health. Two or more fish servings per week are recommended by the American Heart Association [51]. We confirm that two portions of 150 g of fish per week may reduce CVD risk by approximately 8%. Similarly, a daily intake of 50 g (the average size of a drained tuna can) may result in 9% CVD risk reduction. Notably, it seems that higher intake of fish may reduce CVD risk by a quarter or even more, supporting the public health advice to consume more fish.

Our results are supported by numerous experimental studies and related meta-analyses and reviews [6,12,13,43,44]. Moreover, there is a consistent body of evidence on the underlying mechanisms that support the observed reduced CVD risk in relation to fish intake. The mechanistic interpretation of the association between high fish intake and CVD risk reduction lies mainly in the effect of the polyunsaturated fats contained in fish, especially in fatty fish [43,44]. It is suggested that DHA and EPA, which are found mainly in seafood and fatty fish, may play a major role in reducing CVD risk [12,52]. Increased DHA and EPA in cell membrane phospholipids are associated with a reduction of inflammation markers and platelet aggregation [52]. In general, benefits to many other CVD risk factors, such as blood pressure and cardiovascular function, might be linked to ω-3 PUFAs [53,54,55]. Also, fish proteins may contribute to the observed protective role of fish, due to their possible anti-inflammatory effect (measured as a reduction in plasma CRP levels, compared with proteins from land animals). The possibility that a component of this protective effect is due to the displacement of foods with a less favourable health effect profile should also be considered [56]. It is interesting to note that the overall protective effect observed for fish consumption also allows us to conclude that the favourable contribution of long-chain omega-3 fatty acids and of other components of interest evidently outweighs the possible adverse effects associated with the heavy metals, toxic compounds and microplastics that are now sometimes identified in fish.

Our study has numerous strengths. First, this is a systematic synthesis of the most recent evidence from prospective cohort studies conducted using cutting-edge methodology. Our results are supported by a large set of data and number of CVD events. Our sensitivity analysis and stratification analysis demonstrated the robustness of our results with respect to the effect of outlier results, scientific quality and many methodological factors. Second, our study does not only represent an update of the scientific evidence accumulated so far but includes novel results on the association between fish intake and cardiovascular risk by evaluating different CVD outcomes and types of fish intake. Finally, we conducted a non-linear meta-regression analysis to provide quantitative information about the dose–response relationship between fish intake and cardiovascular risk. From this perspective, our work is innovative, as it confirms and extends existing scientific evidence. This work also has several limitations. First, probably because the studies used are from different sources and settings, a moderate to large between-study heterogeneity was demonstrated. This could have affected our analysis, resulting in overdispersed estimates and false-negative results. However, we believe this limitation is minor as we showed that excluding certain studies reduced the between-study heterogeneity without changing the risk estimates. Also, the possibility of false-negative results could have limited some of our stratified analyses, especially those with less than four included studies. However, we were able to detect up to a 5% statistically significant risk reduction. The detection of smaller risks would not be of any practical importance in this context. Second, it must be acknowledged that a high fish intake may result in the accumulation of toxic substances, such as heavy metal ions and mercury, micro plastics and other types of water pollutants. However, according to our analysis, it seems that any intake of such substances in relation to fish intake does not affect CVD risk. From this perspective, it seems that eating fish is beneficial to reducing CVD risk, despite a likely correlation between fish intake and mercury levels [57,58]. We cannot exclude that the correlation between high intake of fish and toxic substances due to pollution may have increased the risk of other diseases, such as cancer or neurodevelopmental diseases. More evidence is needed to fully disentangle the complex association between fish intake, ocean and water pollution and human health as a whole [59].

## 5. Conclusions

Fish consumption, especially fatty fish intake, is beneficial to cardiovascular health, and it should be promoted through public health policies. A small serving of fish a day, or the recommended two portions a week, may reduce the risk of fatal and non-fatal CVD outcomes by approximately 10%. Up to 30% reduced CVD risk can be achieved by eating a full portion of fish a day.

## Figures and Tables

**Table 1:** Characteristics of the included studies.

First Author, Year Country Cohort	AGE (Range) Men (%)	Sample Size Person-Years Follow-Up (Years)	Cardiovascular Events	Adjusting Factors	Sex: Outcome RR (95% CI)	NOS
Amiano et al., 2015 [23] Spain EPIC	49.6 (20–69) 37.8%	41,020 566,076 13.8	STR: 674	AGE, CNT, BMI, WST, SMK, PAC, EDU, ALC, NRG, FOD, DRG, HYP, HPL, CVD, MNP, HRT, OCT	M:STR 0.77 (0.57; 1.16) W:STR 1.07 (0.68; 1.69) M:iSTR 1.13 (0.68; 1.88) W:iSTR 1.31 (0.69; 2.47)	8
Atkins et al., 2014 [24] UK BRHS	68.2 (60–79) 100%	3328 37,606 11.3	CVD *: 327 CVD: 582 CHD: 307	AGE, NRG, SMK, ALC, PAC, SES, BMI, HDL, SBP, DIA, CRP, VWD	M+W:CVD * 0.66 (0.36; 1.21) M+W:CVD 0.85 (0.52; 1.37) M+W:CHD 0.67 (0.37; 1.21)	7
Bellavia et al., 2016 [25] Sweden SMC + CSM	60.1 (45–83) 53.2%	72,522 1,232,874 17.0	CVD *: 4899	AGE, BMI, PAC, SMK, ALC, EDU, NRG, FOD	M+W:CVD * 0.85 (0.76; 0.94)	6
Bonaccio et al., 2017 [26] Italy Moli-sani	54.7 (SD 11) 46.0%	20,969 90,886 4.3	CVD: 353 CHD: 287 STR: 66	AGE, SEX, NRG, EDU, SMK, DRG, DIS, GLU, LDL, INF	M+W:CVD 0.60 (0.40; 0.90) M+W:CHD 0.60 (0.38; 0.94) M+W:STR 0.62 (0.26; 1.51)	8
Gammelmark et al., 2016 [27] Denmark DDCHS	56.2 (50–64) 49.2%	57,053 969,901 17.0	MI: 3028	SMK, BMI, WST, PAC, ALC, EDU, MNP, DIA, HYP, HPL, NRG, FOD	M:MI:FF 0.93 (0.81; 1.07) M:MI:LF 1.12 (0.97; 1.29) W:MI:FF 0.86 (0.69; 1.08) W:MI:LF 0.99 (0.79; 1.24)	7
Hengeveld et al., 2018 [28] The Netherlands EPIC	48.7 (20–70) 25.2%	34,033 612,594 18.0	STR: 753 hSTR: 220 iSTR: 413 CHD: 2134 MI: 693 CVD *: 540	AGE, SEX, PAC, SMK, EDU, BMI, ALC, NRG, SFA, TFA, FOD, FIB	M+W:STR 0.91 (0.79; 1.05) M+W:hSTR 0.79 (0.60; 1.03) M+W:iSTR 0.87 (0.72; 1.05) M+W:CHD 1.03 (0.94; 1.12) M+W:MI 0.97 (0.83; 1.13) M+W:CVD * 0.94 (0.80; 1.10)	8
Key et al., 2019 [29] Europe EPIC	57.1 (SD 8.3) 26.0%	409,885 5,164,551 12.6	CVD *: 7198	AGE, SMK, DIA, HYP, HPL, PAC, WRK, EDU, BMI, ALC, NRG, FOD, FIB	M+W:CVD *:FF 0.92 (0.86; 0.99) M+W:CVD *:LF 1.02 (0.94; 1.11)	8
Kobayashi et al., 2020 [30] Japan JPHC	56.3 (45–74) 46.6%	79,904 1,190,570 14.9	CVD *: 2942	AGE, CNT, BMI, ALC, NRG, SMK, PAC, FOD, BEV, WRK	M:CVD * 0.83 (0.69; 1.00) W:CVD * 0.94 (0.75; 1.19)	7
Kondo et al., 2019 [31] Japan NNSJ	50.0 (30–79) 43.9%	9115 223,771 24.6	CVD *: 1070	AGE, SEX, SMK, NRG	M+W:CVD * 0.72 (0.56; 0.91)	6
Kuhn et al., 2013 [32] Germany EPIC	50.6 (35–65) 42.0%	48,315 393,556 8.1	MI+MI *: 605 MI: 488 MI *: 117 STR: 525 iSTR: 407 hSTR: 95	AGE, CNT, SEX, NRG, ALC, BMI, WST, PAC, EDU, SMK, DIA	M+W:MI+MI * 0.84 (0.66; 1.08) M+W:MI 0.78 (0.59; 1.03) M+W:MI * 1.18 (0.68; 2.06) M+W:STR 0.96 (0.73; 1.26) M+W:iSTR 0.87 (0.64; 1.19) M+W:hSTR 1.46 (0.77; 2.78)	8
Kulezic et al., 2019 [33] Sweden MDCS	57.8 (SD 7.4) 37.7%	26,010 781,417 21.7	PAD: 1122	AGE, SEX, NRG, DAM, SEA, ALC, PAC, SMK, BMI, EDU, FOD	M+W:PAD 0.92 (0.81; 1.04)	7
Mohan et al., 2021 [34] World PURE	50.6 (SD 10.0) 41.7%	147,541 1,342,623 9.1	CVD: 8201 MI: 3806 STR: 3925 CVD *: 3102	AGE, SEX, CNT, BMI, EDU, ALC, PAC, DIA, CNC, DRG, FOD, NRG	M+W:CVD 0.95 (0.86; 1.04) M+W:MI 0.90 (0.78; 1.04) M+W:STR 0.95 (0.83; 1.08) M+W:CVD * 0.94 (0.80; 1.10)	6
Mohan et al., 2021 [34] World OTT	66.5 (SD 7.3) 70.3%	31,491 141,710 4.5	CVD: 5182 MI: 1552 STR: 1395 CVD *: 2265	AGE, SEX, CNT, BMI, EDU, ALC, PAC, DIA, CNC, DRG, FOD, NRG, TRT	M+W:CVD 0.91 (0.81; 1.03) M+W:MI 0.86 (0.69; 1.06) M+W:STR 1.25 (1.00; 1.58) M+W:CVD * 0.80 (0.67; 0.96)	6
Mohan et al., 2021 [34] World ORIGIN	63.6 (SD 7.8) 65.0%	12,422 77,016 6.2	CVD: 2020 MI: 591 STR: 533 CVD *: 1135	AGE, SEX, CNT, BMI, EDU, ALC, PAC, DIA, CNC, DRG, FOD, NRG, TRT	M+W:CVD 0.87 (0.76; 1.01) M+W:MI 1.16 (0.90; 1.49) M+W:STR 0.82 (0.62; 1.09) M+W:CVD * 0.78 (0.64; 0.94)	6
Nahab et al., 2020 [35] USA REGARDS	63.7 (NR) 41.0%	16,479 83,431 5.1	CVD: 700 MI: 440 iSTR: 265 CVD *: 291	AGE, SEX, CNT, ETH, EDU, PAC, SMK, DIS, DRG, NRG, DIA, SBP, HPL	M+W:CVD 1.63 (1.11; 2.40) M+W:CVD 1.09 (0.78; 1.52) M+W:MI 1.48 (0.90; 2.43) M+W:MI 0.87 (0.56; 1.35) M+W:iSTR 1.83 (0.99; 3.39) M+W:iSTR 1.58 (0.95; 2.63) M+W:CVD * 0.74 (0.35; 1.55) M+W:CVD * 1.46 (0.87; 2.45)	5
Owen et al., 2016 [36] Australia AusDiab	51.5 (SD 11.2) 44.8%	11,207 141,208 12.6	CVD *: 277	AGE, SEX, CVD, SMK, NRG, PAC, EDU	M:CVD * 0.69 (0.40; 1.20) W:CVD * 0.85 (0.42; 1.73)	6
Rhee et al., 2017 [37] USA WHS	54.6 (SD 7.1) 0%	39,392 713,559 18.1	CVD: 1941	TRT, AGE, BMI, SMK, ALC, PAC, OCT, HRT, VIT, NRG, FCD, HYP, CHL, DIA	M+W:CVD 1.00 (0.88; 1.16)	6
Takata et al., 2013 [38] China SHS	53.9 (SD 9.3) 45.5%	80,578 656,662 8.2	CVD *: 1789 iHD *: 476 iSTR *: 404 hSTR *: 460	AGE, NRG, INC, WRK, EDU, COM, PAC, FOD, SMK, ALC	M+W:CVD * 0.86 (0.70; 1.05) M+W:iHD * 1.02 (0.74; 1.41) M+W:iSTR * 0.63 (0.41; 0.94) M+W:hSTR * 0.90 (0.43; 1.07)	7
Van den Brandt et al., 2019 [39] The Netherlands NCS	61.4 (55–62) 47.9%	3202 8701 2.2	CVD *: 733	AGE, SEX, SMK, HYP, DIA, HGT, BMI, PAC, EDU, ALC, FOD, HRT, VIT	M+W:CVD * 1.45 (1.20; 1.74)	5
Zhang et al., 2018 [40] USA NIH-AARP	62.1 (RIQ 57–66) 57.1%	421,309 6,070,000 14.4	CVD *: 29,648	AGE, BMI, ETH, EDU, MAR, SMK, ALC, NRG, FOD, PAC, VIT, DRG, DIA, HYP, CHL	M:CVD * 0.90 (0.85; 0.94) W:CVD * 0.90 (0.83; 0.97)	7

Legend: SD: Standard deviation, NR: Not reported, RIQ: Interquartile range; Cardiovascular events: CVD: All cardiovascular events, HD: Heart disease, MI: Myocardial infarction, STR: Stroke, PAD: Peripheral artery disease, (i): ischaemic, (h): haemorrhagic, (*): mortality due to, FF: Fatty fish, LF: Lean fish; M: Men, W: Women; Adjusting factors: AGE: Age, ALC: Alcohol use, BEV: Specific beverages (coffee, green tea, etc.), BMI: Body mass index, CHL: Cholesterol, CNC: Cancer at baseline, CNT: Centre, area or geographical feature, COM: Any comorbidity, CRP: C-reactive protein, CVD: Baseline presence of any cardiovascular disease, DAM: Diet assessment method, DIA: Diabetes, DIS: Any type of diet score, DRG: Drug use, EDU: Education, ETH: Ethnicity, FCD: Family history of any cardiovascular disease, FIB: Dietary fibre, FOD: Various types of food (vegetables, read meat, etc.), GLU: Blood glucose, HDL: High-density lipoprotein, HGT: Height, HYP: Hypertension, HPL: Hyperlipidaemia, HRT: Hormone replacement therapy, INC: Income, INF: Inflammation status, LDL: Low-density lipoprotein, MAR: Marital status, MNP: Menopausal status, NRG: Energy intake, OCT: Oral contraceptive, PAC: Physical activity, SBP: Systolic blood pressure, SEA: Season, SES: Socioeconomic status, SEX: Sex, SFA: Saturated fatty acids, SMK: Smoking, TFA: Trans fatty acids, TRT: Treatment group in nested RCT studies, VIT: Vitamin supplements, VWD: Von Willebrand disease, WRK: Employment status or occupation, WST: Waist circumference.

**Table 2:** Stratified analyses and assessment of the heterogeneity determinants.

	#RRs	RR (95% CI)	I^2^ (%)	I^2^_Res._ (%)	* *p*-Value
Age					
<55 years	11	0.95 (0.89; 1.01)	78.3	74.0	0.241
≥55 years	6	0.86 (0.76; 0.98)	56.8		
Sex					
Men	7	0.91 (0.89; 1.01)	43.5	19.60	0.888
Women	7	0.86 (0.76; 0.98)	0.0		
Geographical area					
Europe	7	0.96 (0.86; 1.06)	81.2	74.6	0.807
Asia-Pacific	4	0.95 (0.78; 1.14)	78.3		
USA	3	0.98 (0.75; 1.27)	79.5		
World	3	0.89 (0.83; 0.96)	41.5		
Publication year					
<2019	8	0.90 (0.84; 0.97)	55.0	73.4	0.340
≥2019	9	0.95 (0.87; 1.04)	80.4		
Sample size					
<30,000	8	0.91 (0.75; 1.09)	83.9	74.0	0.918
≥30,000	9	0.93 (0.89; 0.96)	43.6		
Adjusting factors					
<8 factors	4	0.84 (0.77; 0.91)	0.0	70.3	0.134
≥8 factors	13	0.95 (0.90; 1.01)	75.2		
With NRG	15	0.91 (0.87; 0.95)	59.3	64.2	0.031
Without NRG	2	1.08 (0.57; 2.05)	86.5		
With FOD	11	0.93 (0.88; 0.98)	73.9	74.0	0.547
Without FOD	6	0.86 (0.69; 1.06)	74.4		
With CVD	11	0.97 (0.91; 1.03)	77.2	70.1	0.075
Without CVD	6	0.85 (0.79; 0.92)	21.4		
NOS score					
<6	2	1.37 (1.19; 1.57)	0.0	46.5%	<0.001
≥6	15	0.90 (0.87; 0.94)	48.3		
<7	9	0.95 (0.85; 1.06)	81.7	74.1	0.469
≥7	8	0.92 (0.88; 0.97)	50.1		

Notes. #RRs: Number of estimates included, RR (95% CI): Meta-analytical estimate of relative risk and 95% confidence limits, I^2^_Res_: Residual heterogeneity after meta-regression, *: *p*-value for the comparison of pooled estimates by strata, NRG: Estimates adjusted for energy intake, FOD: Estimates adjusted for different types of food intake, CVD: Estimates adjusted for any type of baseline CVD condition, type II diabetes, hypertension or hyperlipidaemia.

## Data Availability

Data are available on request from the corresponding author.

## Supplementary Materials

The following supporting information can be downloaded at: https://www.mdpi.com/article/10.3390/nu15214539/s1, Figure S1: Publication bias assessment. Supplementary Material S1.txt: Search string.

## Abbreviations

CHD	Coronary heart disease
CVD	Cardiovascular disease
DALYs	Disability-adjusted life years
DHA	Docosahexaenoic acid
EPA	Eicosapentaenoic acid
FF	Fat fish
HD	Heart disease
LF	Lean fish
MeSH	Medical Subject Headings
MI	Myocardial infarction
NCD	Non-communicable disease
NOS	Newcastle-Ottawa Scale
NR	Not reported
PAD	Peripheral artery disease
PECO(ST)	P: Population E: Exposure C: Comparison O: Outcome S: Study type T: Time of publication
PRISMA	Preferred Reporting Items for Systematic Reviews and Meta-Analyses
PUFA	Polyunsaturated fatty acids
RIQ	Interquartile range
RR	Relative risk
CI	95% confidence interval
SD	Standard deviation
STR	Stroke
hSTR	Haemorragic stroke
iSTR	Ischemic stroke
