# Supplementary figures and images for: Fish Intake in Relation to Fatal and Non-Fatal Cardiovascular Risk: A Systematic Review and Meta-Analysis of Cohort Studies
Source: Nutrients. 2023 Oct 26;15(21):4539. doi: 10.3390/nu15214539 (PMC10647504; doi:10.3390/nu15214539)

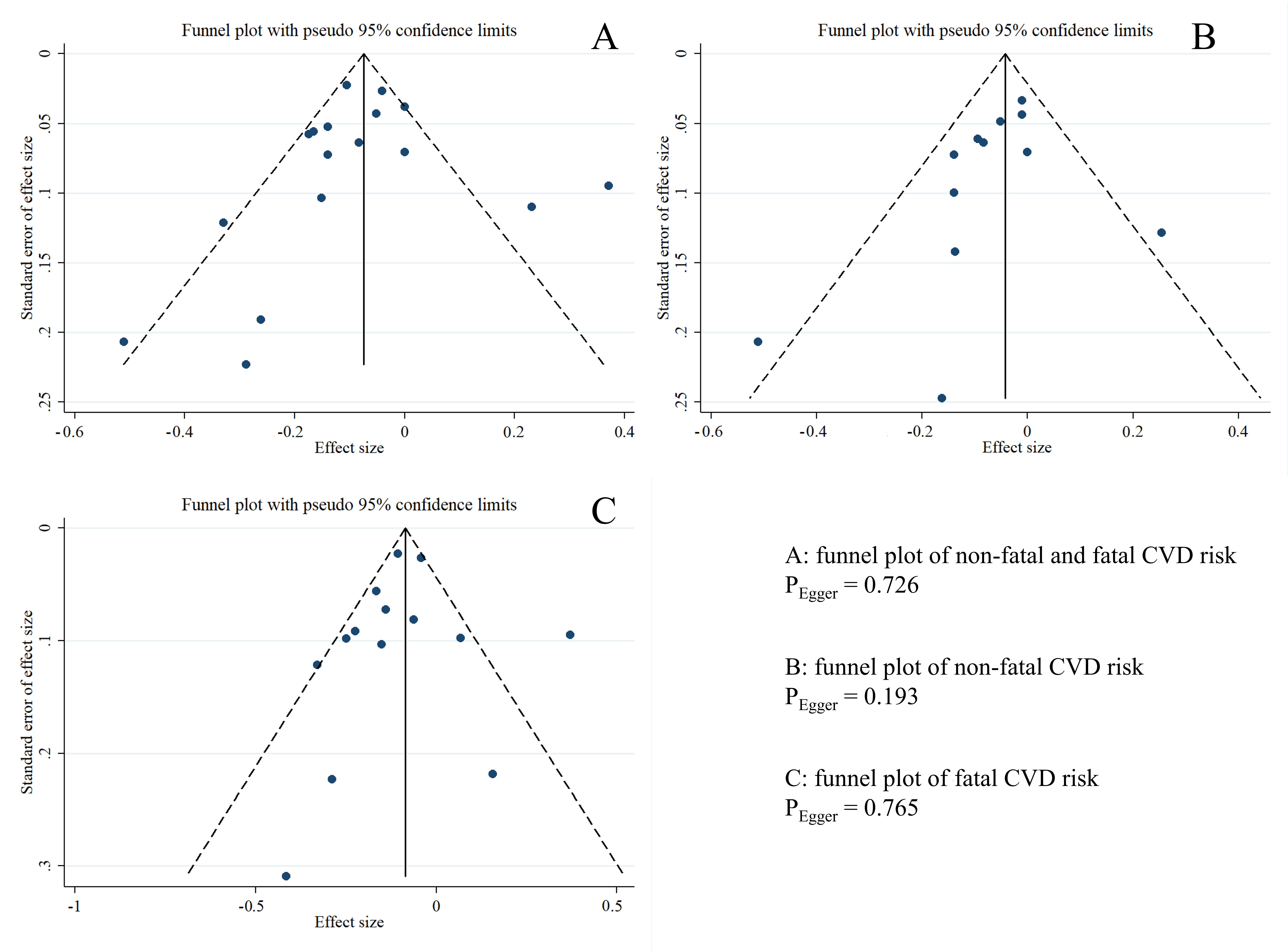

Supplement: Supplementary file 1 [file nutrients-15-04539-s001.zip › Supplementary Figure S1.tif]
